# Neuroimaging Features of San Luis Valley Syndrome

**DOI:** 10.1155/2015/748413

**Published:** 2015-09-06

**Authors:** Matthew T. Whitehead, Bonmyong Lee

**Affiliations:** ^1^Department of Radiology, Children's National Medical Center, Washington, DC 20010, USA; ^2^Department of Radiology, Johns Hopkins Medical Institute, Baltimore, MD 21287, USA

## Abstract

A 14-month-old Hispanic female with a history of double-outlet right ventricle and developmental delay in the setting of recombinant chromosome 8 syndrome was referred for neurologic imaging. Brain MR revealed multiple abnormalities primarily affecting midline structures, including commissural dysgenesis, vermian and brainstem hypoplasia/dysplasia, an interhypothalamic adhesion, and an epidermoid between the frontal lobes that enlarged over time. Spine MR demonstrated hypoplastic C1 and C2 posterior elements, scoliosis, and a borderline low conus medullaris position. Presented herein is the first illustration of neuroimaging findings from a patient with San Luis Valley syndrome.

## 1. Introduction

San Luis Valley syndrome (SLVS) is a rare chromosomal inversion disorder that prevalently affects individuals of Hispanic origin. The name is derived from a presumed founder effect traced to the San Luis Valley in the southwestern United States. SLVS is caused by deletion of the terminal part of the short arm of chromosome 8 (8pter-p23.1) together with duplication of the terminal part of the long arm of chromosome 8 (8q22.1-qter) [1, 2].

Characteristic features of SLVS include facial dysmorphia, congenital heart disease, and genitourinary abnormalities [3]. Developmental delay, intellectual disability, and seizures are well-documented neurologic manifestations [3–5]. However, little is known about potential structural brain defects resulting from SLVS. Brain imaging and histopathological abnormalities have rarely been described; central nervous system imaging abnormalities have not been previously illustrated. We present MRI features in the brain and spine from a 14-month-old patient with San Luis Valley syndrome.

## 2. Case Presentation

A neonatal Hispanic female was referred to the department of genetics for evaluation of facial dysmorphism, congenital heart disease, and a two-vessel umbilical cord suggestive of an underlying syndrome. She was the product of an uncomplicated pregnancy, born at term. Karyotype analysis was compatible with San Luis Valley syndrome (46, XX, Rec (8) Dup (8Q) Inv (8) p23.1 q22.1). Past medical history was notable for complex cardiac deformities including double-outlet right ventricle, ventricular septal defect, subvalvular pulmonic stenosis, pulmonary arterial hyperplasia, restrictive cardiomyopathy, and an aberrant retroesophageal right subclavian artery. The patient had previously undergone cardiac surgery for tunnel repair and infundibular resection. She had facial dysmorphism typifying the San Luis Valley syndrome including hypertelorism, frontal bossing, wide-spaced teeth, elevated palate, and down-turned corners of the mouth. She also had nystagmus, astigmatism, intermittent esotropia, and refractive errors. However, external, anterior, retinal, and dilated fundoscopic exams were normal. At 14 months of age, she was referred to our imaging service to have a brain MRI for developmental delay.

**Figure 1:**
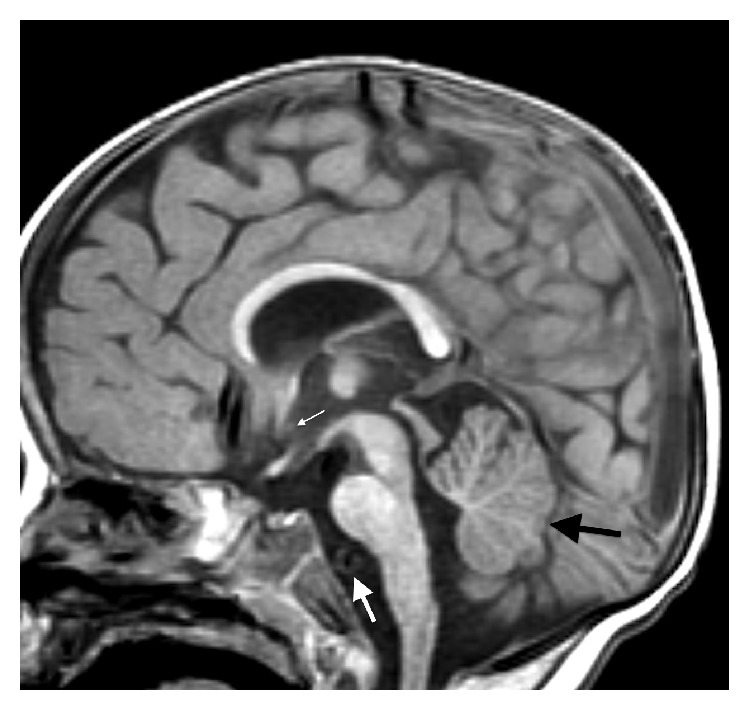
Sagittal SPGR T1WI (repetition time msec/echo time msec/inversion time msec, 13/5/500) showing multiple midline brain abnormalities including a thin corpus callosum, brainstem hypoplasia, vermian hypoplasia (black arrow), and a subtle interhypothalamic adhesion (small white arrow). There is also vertebrobasilar ectasia, abutting and deforming the basis pontis (large white arrow).

**Figure 2:**
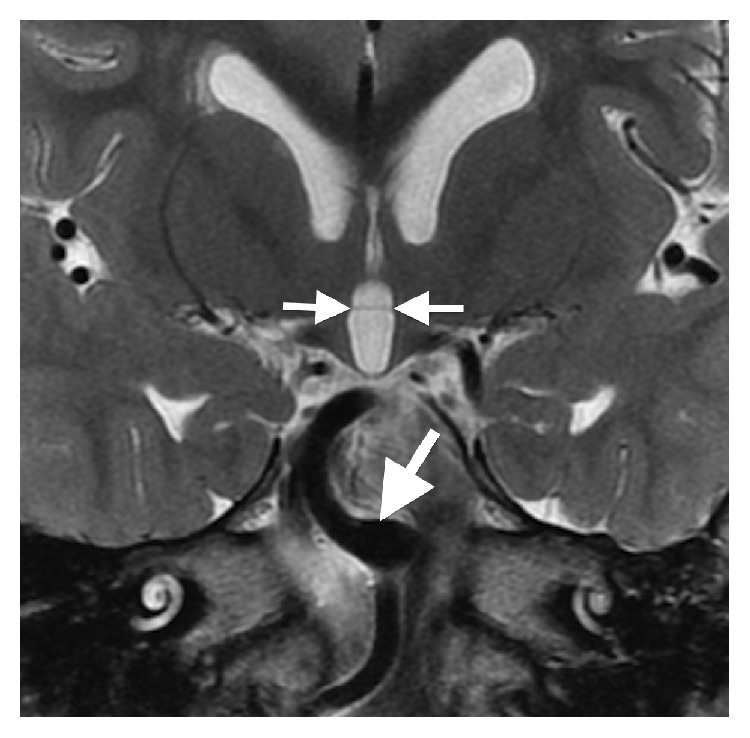
Coronal T2WI (repetition time msec/echo time msec, 2767/102) depicting a horizontally oriented band of tissue connecting the hypothalami to one another, an interhypothalamic adhesion (small arrows). The basilar artery is tortuous and ectatic (large arrow).

**Figure 3:**
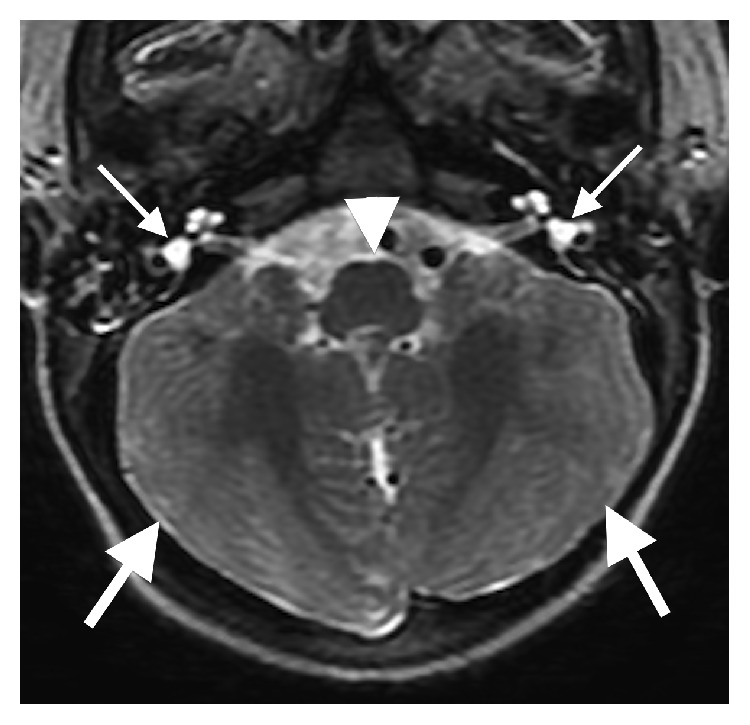
Axial T2-weighted fast spin echo image (repetition time msec/echo time msec, 6083/108) demonstrating irregular cerebellar fissuration/foliation consistent with dysplasia (large arrows), enlarged inner ear vestibules representing dysplasia (small arrows), and malformation of the medulla oblongata with underdevelopment of the median sulcus and absent preolivary sulci (arrowhead). The vermis is not present on this transaxial section as it should be, consistent with vermian hypoplasia.

**Figure 4:**
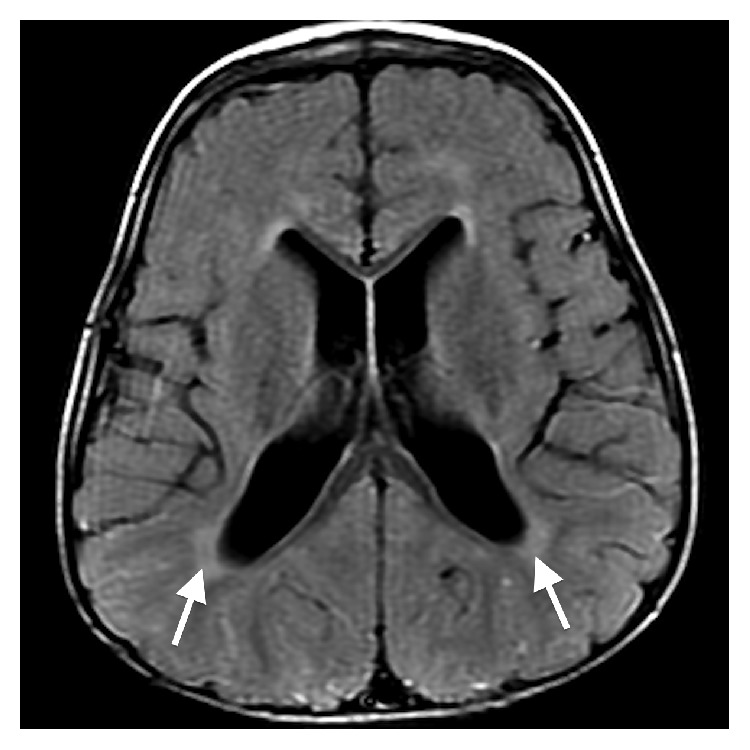
Axial T2 FLAIR image (repetition time msec/echo time msec/inversion time msec, 10002/135/2200) through the lateral ventricles depicting deep/paraventricular white matter hyperintensity consistent with gliosis from old injury (arrows). Diminished cerebral white matter volume is present with decreased white matter depth and mild ventriculomegaly.

The initial brain MR revealed multiple midline abnormalities including corpus callosum dysgenesis, anterior commissure hypoplasia, an interhypothalamic adhesion, and brainstem and vermian hypoplasia/dysplasia (Figures 1 and 2). The medulla oblongata was dysmorphic with hypoplasia of the median sulcus and absence of the preolivary sulci, the cerebellum was dysplastic with irregular cerebellar fissuration/foliation, and the inner ear vestibules were patulous and dysmorphic (Figure 3). In addition, the major intracranial arteries of the anterior and posterior circulation were ectatic and mildly tortuous; vertebrobasilar dolichoectasia caused mass effect on the pons (Figures 1 and 2). There was also cerebral white matter volume loss or hypoplasia and multifocal hyperintense cerebral white matter lesions compatible with gliotic changes from old injury (Figure 4). Additional brain MR abnormalities not illustrated here included bilateral incomplete hippocampal inversion, old parenchymal microhemorrhages, mild right maxillary sinus hypoplasia, and microphthalmia with short globe anteroposterior dimensions.

**Figure 5:**
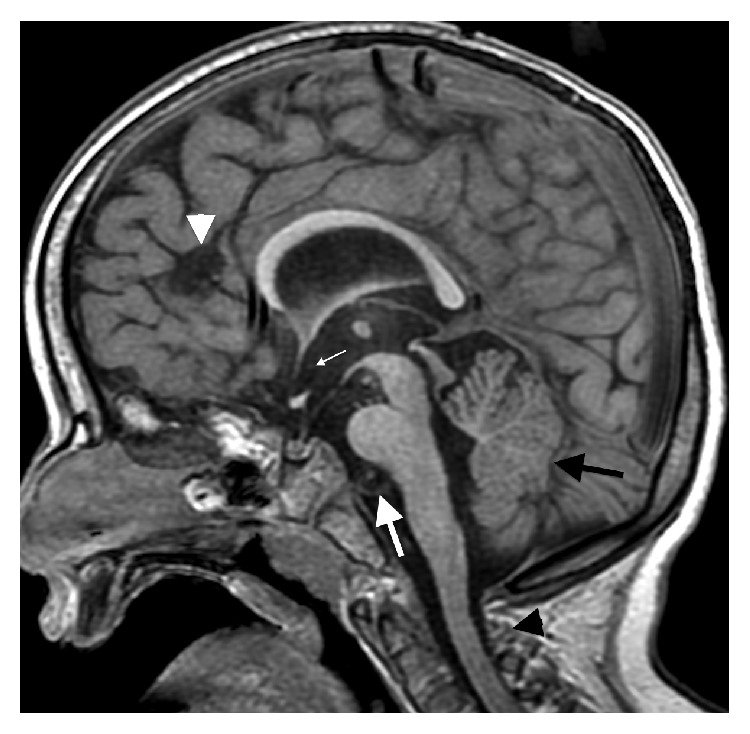
Sagittal SPGR T1WI (repetition time msec/echo time msec/inversion time msec, 13/5/500) redemonstrating multiple midline brain abnormalities including a thin, dysgenetic corpus callosum, brainstem hypoplasia, vermian hypoplasia (black arrow), and an interhypothalamic adhesion (small white arrow). A newly visible extra-axial hypointense structure is centered in the anterior cingulate sulci representing an epidermoid (white arrowhead). Vertebrobasilar ectasia is again shown, abutting and deforming the basis pontis (large white arrow). Normal C1 and C2 posterior elements are not identified on this midline sagittal image, consistent with hypoplasia (black arrowhead).

**Figure 6:**
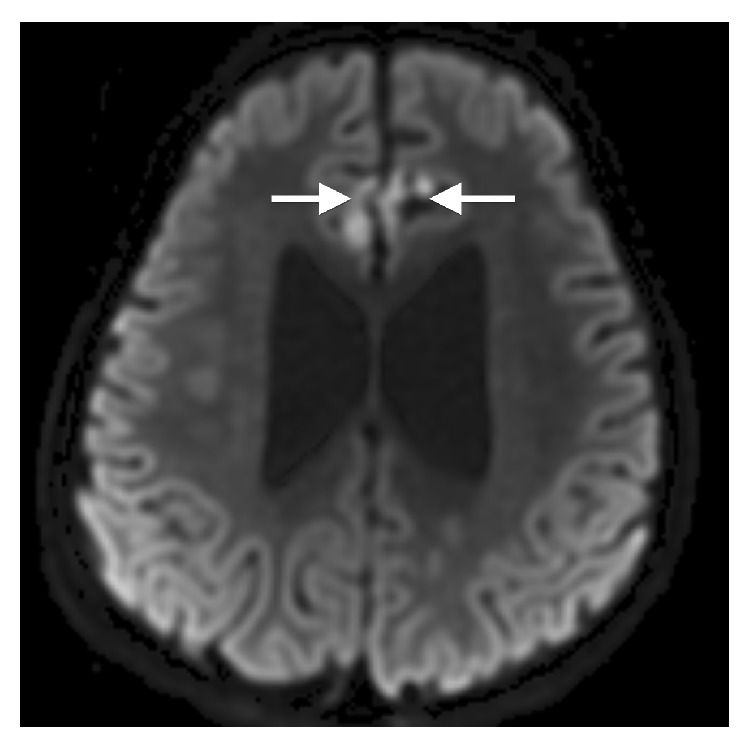
Axial diffusion weighted image (repetition time msec/echo time msec, 10000/82; *b* = 1000 msec) demonstrating hyperintensity compatible with reduced diffusion associated with an interhemispheric epidermoid between the frontal lobes (arrows).

**Figure 7:**
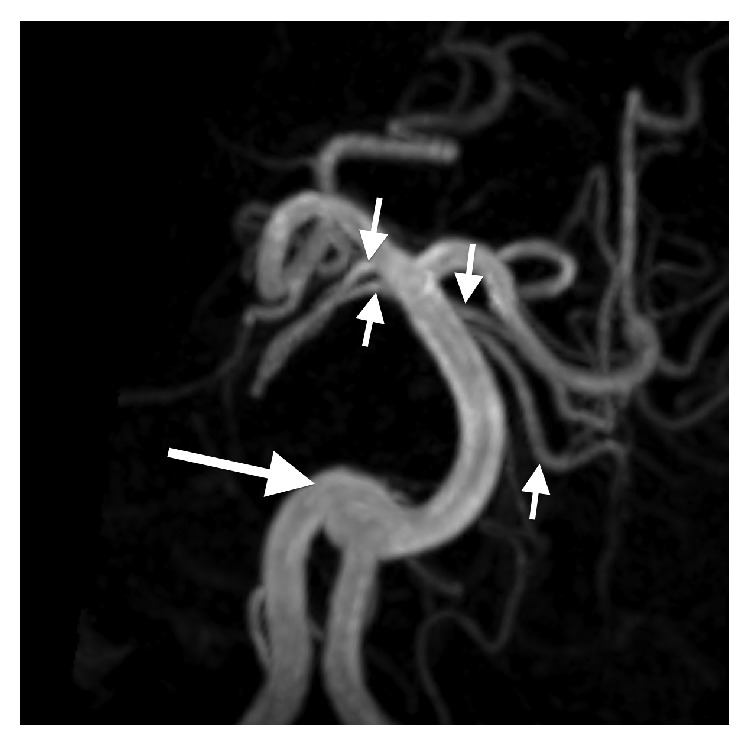
Maximum intensity projection image from a 3D time-of-flight MR angiogram of the vertebrobasilar system displayed in the coronal plane (repetition time msec/echo time msec, 21/2) demonstrating vertebrobasilar dolichoectasia (large arrow) and duplication of the superior cerebellar arteries (small arrows).

At 5 years, she developed seizures with episodes leading to apnea, desaturations, and bradycardia. Repeat brain MR demonstrated, in addition to the previously seen midline defects, an epidermoid cyst interposing the frontal lobes in the anterior interhemispheric region (Figures 5 and 6). No other significant pathologic change occurred. Myelination progressed normally. MRA revealed a duplicated right anterior cerebral artery A1 segment and duplicated superior cerebellar arteries. MRA also depicted arterial tortuosity and ectasia including stable dolichoectasia of the vertebrobasilar system (Figure 7). A total spine MR performed concurrently showed the C1 and C2 posterior elements to be hypoplastic (Figure 5), 6 nonrib bearing lumbar type vertebral bodies, a mild thoracolumbar dextroscoliosis, paraspinal muscular atrophy, and a borderline low termination of the conus medullaris at the L2-L3 level.

Currently at 7 years, the patient has medically refractory severe complex partial epilepsy. Her language and motor skill developmental milestones are markedly delayed. She has hyperopia and nystagmus. Eustachian tube dysfunction has led to intermittent middle ear infections. She ultimately underwent percutanous nephrostomy for lithotripsy. The most recent brain MRI failed to demonstrate any significant interval change.

## 3. Discussion

San Luis Valley syndrome is a genetic disease caused by a recombinant chromosome 8 pericentric inversion [4]. Multiple bodily systems are affected, especially cardiac; over 90% have congenital heart defects [3]. As in this case, developmental delay, intellectual disability, and seizures are well-known neurologic manifestations. Previous reports describe variable brain involvement, ranging from normal to abnormal in appearance (Table 1). However, the neuroimaging findings of San Luis Valley syndrome have not been previously illustrated in the literature. The largest SLVS study described brain ultrasound, CT, and MRI findings including ventriculomegaly, volume loss, delayed myelination, and corpus callosum dysgenesis [3]. Vera-Carbonell and colleagues reported isolated mild ventriculomegaly on fetal MRI and postnatal ventricular asymmetry, dilated temporal horn, and colpocephaly in a Moroccan female patient with a recombinant chromosome 8 defect that differed from classical SLVS (8p23.2pter deletion and 8q22.3-qter duplication) [6]. Brains from patients with San Luis Valley syndrome range from being normal to markedly abnormal at autopsy. Occipital encephalocele, hydrocephalus, and prominence of the pons have all been documented [7]. Sequela of hypoxic injury from presumed congenital heart disease can also be seen histologically [7].

Our patient had multiple central nervous system abnormalities predominantly affecting midline structures. Specifically, commissural white matter fibers traversing the corpus callosum and anterior commissure were hypoplastic and the corpus callosum was dysmorphic consistent with corpus callosum dysgenesis. An interhypothalamic adhesion was present, a marker for defective midline brain formation and potentially a form fruste holoprosencephaly [11, 12]. The cerebellum and brainstem were hypoplastic and dysplastic. A unique malformation of the medulla oblongata was manifested by underdevelopment of the anterior median sulcus and aplastic preolivary sulci. An epidermoid cyst was present between the frontal lobes. Interestingly, this lesion was not visible on the first MRI. However, because epidermoids are congenital, the epidermoid cyst was likely present but below the resolution of detection and grew to become conspicuous over time.

MR images from our patient also exhibited bilateral vestibular dysplasia. The inner ear vestibules house the utricles and saccules that influence the sensation of balance equilibrium. Structural and/or functional vestibular abnormalities have not been previously described to the best of our knowledge. The majority of SLVS patients have conductive and/or sensorineural hearing loss [3]. Our patient had no documented vestibular symptoms or sensorineural hearing loss. Chronic eustachian tube dysfunction did cause intermittent middle ear infections in our patient, however. The recurrent otitis media was successfully treated with tympanostomy tubes.

We also noted mild bilateral microphthalmia in our patient. The globes were symmetrically small in the anteroposterior dimensions. This may account for the patient's hyperopia. Ophthalmologic abnormalities have been found in a majority of patients with San Luis Valley syndrome [3]. These include strabismus, nystagmus, refractory errors, optic nerve pallor, enlarged optic disks, and retinal changes.

The intracranial arterial flow voids were mildly tortuous and ectatic in our patient. There was notable dolichoectasia of the basilar artery causing mass effect on the basis pontis. Numerous cerebral white matter lesions were consistent with gliosis from prior injury. A few cerebral and cerebellar old microhemorrhages were also present. The cerebral white matter volume was decreased. Collectively, these findings were most likely related to complications of congenital heart disease and treatment. It is unclear to what extent the underlying genetic defect may have predisposed or contributed to these changes. Though occasionally seen as a normal variation, duplication of the superior cerebellar arteries and right anterior cerebral artery A1 segment could indicate an underlying intracranial vascular tree malformation in concert with the more generalized arterial tortuosity, arterial ectasia, and congenital heart disease.

Concerning the spine, we found hypoplasia of the C1 and C2 posterior elements, an L6 vertebral body, mild scoliosis, paraspinal muscular atrophy, and borderline low termination of the conus medullaris. There were no documented symptoms of tethered spinal cord syndrome. There are no previous studies with cross-sectional imaging correlation demonstrating spinal abnormalities in patients with San Luis Valley syndrome. However, Sujansky and colleagues reported what was described as a noncongenital progressive neuromuscular scoliosis in 13 of 20 SLVS patients that underwent spine radiography [3]. Five of 20 SLVS patients had scoliosis on either chest and/or spine radiography according to Williamson and Clericuzio [5]. The mild thoracolumbar scoliosis and paraspinal atrophy in our patient was consistent with a neuromuscular type scoliosis. There were no detectable thoracolumbar spinal segmentation anomalies.

## 4. Conclusion

We present neuroimaging abnormalities associated with San Luis Valley syndrome. Multiple midline anomalies including commissural dysgenesis, interhypothalamic adhesion, epidermoid cyst, and brainstem and cerebellar dysgenesis/hypogenesis were the most striking features.

## Figures and Tables

**Table 1:** Brain abnormalities in patients with SLVS Rec(8) and duplication 8q.

Author	*N*	Defect	Method of analysis	Midline defects	BS	CB	Cephalocele	CSF spaces	Myelin	WM lesions	V
Whitehead (our patient)	1	Rec(8)a	MR	CCD HAC IHA	Yes	Yes	No	VM SE	WNL	Yes	Yes

Sujansky et al. [3]	15	Rec(8)a	CT/US (*n* = 15) MR (*n* = 4)	CCD (1/15)	NM	NM	No	VM (13/15) SE (8/13)	Delay (4/4)	NM	NM

Vera-Carbonell et al. [6]	1	Rec(8)b	MR	NM	NM	NM	NM	VM	NM	NM	NM

Williams et al. [7]	9	Rec(8)a (*n* = 4) Rec(8)c (*n* = 1) U (*n* = 4)	Path	CCT U (1/4)	Yes (Pons) U (1/4)	NM	Yes (OE) U (1/4)	Hydro U (1/4)	NM	Yes (HIE) Rec(8a) (2/4) U (2/4)	NM

Nucaro et al. [8]	1	Rec(8)d	MR	NM	NM	NM	NM	NM	NM	PVL	NM

Wheeler [9]	1	Dup(8)a	US	WNL	No	No	No	WNL	NA	No	NA

Concolino et al. [10]	1	Dup(8)b	MR	NM	NM	NM	Yes (FM)	NM	NM	NM	NM

*N* = number of patients, BS = brainstem malformation, CB = cerebellar malformation, WM = white matter, V = vasculopathy, MR = magnetic resonance imaging, CT = computed tomography, US = ultrasound, Path = histopathology, CCD = corpus callosum dysgenesis, CCT = corpus callosum thinning, HAC = hypoplastic anterior commissure, IHA = interhypothalamic adhesion, NM = not mentioned, NA = not assessed, WNL = within normal limits, OE = occipital encephalocele, FM = frontal meningocele, VM = ventriculomegaly, SE = subarachnoid space enlargement, hydro = hydrocephalus, HIE = hypoxic ischemic encephalopathy, PVL = periventricular leukomalacia.

Rec(8)a = Rec(8)dup(8q)inv(8)(p23.1q22.1);

Rec(8)b = Rec(8)dup(8q)inv(8)(p23.2q22.3)mat.ish rec(8)(wcp8þ).mlpa 8psubtel(P036) × 1,8qsubtel(P036) × 3.arr 8p23.3p23.2(1–2,274,223) × 1,8q22.3q24.3(104,430,376–146,364,022) × 3;

Rec(8)c = Rec(8)dup(8q)inv(8)(p23q23);

Rec(8)d = InvDupDel 8p(dup 8p22–p23.1/del 8p23.2-pter);

Dup(8)a = Dup (8)(q23.3q24.21);

Dup(8)b = Dup (8)(q22.2–24.3)(q24.21), 8q22.2 (RP11-102K7 clone-101.2Mb) to 8q24.3 (RP11-120B22 clone-144.3Mb);

U = unknown or unconfirmed.
